# LeasyScan: a novel concept combining 3D imaging and lysimetry for high-throughput phenotyping of traits controlling plant water budget

**DOI:** 10.1093/jxb/erv251

**Published:** 2015-06-01

**Authors:** Vincent Vadez, Jana Kholová, Grégoire Hummel, Uladzimir Zhokhavets, S.K. Gupta, C. Tom Hash

**Affiliations:** ^1^ICRISAT—Crop Physiology Laboratory, Greater Hyderabad, Patancheru 502324, Telangana, India; ^2^Phenospex, Jan Campertstraat 11 / NL-6416 SG Heerlen, The Netherlands; ^3^ICRISAT, Sahelian Center, Pearl Millet Breeding, BP 12404, Niamey, Niger

**Keywords:** Drought, gravimetric transpiration, high-throughput phenotyping, lysimetric platform, multi-discipline, physiology, vapour pressure deficit, 3D laser scanner.

## Abstract

In this paper, we describe the thought process and initial data behind the development of an imaging platform (LeasyScan) combined with lysimetric capacity, to assess canopy traits affecting water use (leaf area, leaf area index, transpiration). LeasyScan is based on a novel 3D scanning technique to capture leaf area development continuously, a scanner-to-plant concept to increase imaging throughput and analytical scales to combine gravimetric transpiration measurements. The paper presents how the technology functions, how data are visualised via a web-based interface and how data extraction and analysis is interfaced through ‘R’ libraries. Close agreement between scanned and observed leaf area data of individual plants in different crops was found (R^2^ between 0.86 and 0.94). Similar agreement was found when comparing scanned and observed area of plants cultivated at densities reflecting field conditions (R^2^ between 0.80 and 0.96). An example in monitoring plant transpiration by the analytical scales is presented. The last section illustrates some of the early ongoing applications of the platform to target key phenotypes: (i) the comparison of the leaf area development pattern of fine mapping recombinants of pearl millet; (ii) the leaf area development pattern of pearl millet breeding material targeted to different agro-ecological zones; (iii) the assessment of the transpiration response to high VPD in sorghum and pearl millet. This new platform has the potential to phenotype for traits controlling plant water use at a high rate and precision, of critical importance for drought adaptation, and creates an opportunity to harness their genetics for the breeding of improved varieties.

## Highlight

We present a new concept combining novel 3D scanning of the plant canopy with seamless assessment of plant water use to measure plant traits influencing the water budget.

## Introduction

In a companion paper we have reviewed the opportunities that imaging technology now offers to the field of plant phenotyping (Vadez *et al.*, 2015—unpublished), in addition to recent reviews (Fiorani and Schurr 2013; Deery *et al.*, 2014; Li *et al.*, 2014). We have also laid out the potential risks and opportunities of these new technologies and argued for the need to have research questions driving the development of phenotyping platforms to target those phenotypes that are the most relevant for target agroecologies. Previous studies have shown that water availability during the grain filling period is absolutely essential for crop production under drought stress (e.g. Zaman-Allah *et al.*, 2011; Vadez *et al.*, 2013a). We have shown that these differences in the pattern of plant water use are explained by traits altering the water budget at vegetative stage and expressing under fully irrigated conditions (e.g. Kholová *et al.*, 2010; Vadez *et al.*, 2013b). In short, these traits revolve around the development of the leaf area (how quick, how large) and canopy conductance aspects (Vadez *et al.*, 2013b). The first part of the paper will briefly recall the rationale for these traits, and how that knowledge has led the thought process behind the development of the LeasyScan platform.

The following section presents the new platform concept, targeted to trait phenotyping based on 3D imaging of the plant leaf area, using a system where plants are in-site/undisturbed and scanners are moved above the plants. A similar approach has been used recently in which light curtain arrays are projected over the plant (Fanourakis *et al.*, 2014). This new platform also combines the principle of monitoring plant water use gravimetrically, described earlier (Vadez *et al.*, 2014, 2015—unpublished), by having pot weight continuously monitored by analytical scales. Many existing platforms worldwide are using a concept where plants are grown in glasshouses, then moved to an imaging cabinet where different images are taken (e.g. Berger *et al.*, 2010; Golzarian *et al.*, 2011). A 3D crop canopy is then reconstructed from several 2D images. In contrast LeasyScan follows a sensor-to-plant-concept, like others (e.g. Granier *et al.*, 2006; Fanourakis *et al.*, 2014), and introduces four principles: (i) it is based on 3D laser triangulation of the crop canopy, using a laser triangulation sensor, which provides 3D images in high resolution since plants cannot be rotated to gather several images from different perspectives; (ii) the scanners are moved above the crop canopy, which allows higher throughput than moving plants to an imaging station; (iii) the platform combines instantaneous measurements of crop canopy growth and plant transpiration; and (iv) the platform is set outdoors and each data point has a time stamp that links it to continuously monitored environmental conditions. When scans are obtained at a high rate [approximately 4600 sectors (i.e. experimental units) scanned ~10–12 times per day] on several parameters per plant, data management becomes a major challenge (Cobb *et al.*, 2013). This section then also presents the web-based interface that is used to visualise the data (HortControl^R^) and the data management tools used to query data from the database and initiate the data analysis process via ‘R’-scripts libraries. Critical planning aspects during the development of a phenotyping platform are also discussed, such as the need to test the technology prior to acquisition and the need for a close user-provider relationship during and after the development of platforms.

The last section presents the principles of the scanning operation and data comparing scan-derived parameters versus destructive observations of leaf area, scanning both individual plants and plants grown at densities reflecting the field conditions. This section presents the visualization of analytical scale measurements and how transpiration data are derived. The last part of this section presents three case studies illustrating some of the potential uses of the LeasyScan platform to target critical phenotypes and underlying biological functions.

## Setting the needs: what traits and then what platform?

### Leaf area development

Traits related to the canopy development are tightly associated to plant water use and are a combination of (i) vigour, i.e. how quickly the leaf area develops (e.g. fig. 2 in Kholová *et al.*, 2014); and (ii) size, i.e. how large the leaf area develops (fig. 1 of Vadez *et al.*, 2013b). Measuring leaf area is of course the rate-limiting step if these phenotypes are to be used in breeding. Therefore, a platform was needed in which leaf area could be assessed non-destructively and at a fairly high frequency (at least once per day). Here we fell in favour of a system that would follow the leaf area of whole plants rather than the leaf development of specific leaves (e.g. Reymond *et al.*, 2003). In addition, we opted for a system in which plants would be cultivated at a density reflecting the field conditions, rather than individual plants. The expansion of leaves is strongly influenced by environmental factors such as vapour pressure deficit or soil water content and sensitivity to either factor share at least a partial genetic basis (Welcker *et al.*, 2011). Therefore, the leaf area that is measured at any point in time is, itself, a consequence of other factors that have prevailed before, and possibly the response to environmental conditions. The platform then included a careful monitoring of environmental conditions (especially temperature, relative humidity, light and wind speed). Earlier work on the effect of VPD on the expansion of leaf 6 in maize compared leaf expansion during night and day periods (e.g. Reymond *et al.*, 2003) and for that the leaf area was measured continuously. This is difficult in a platform where plants move to an imaging cabinet, which limits the throughput. Therefore, we designed a platform using a reversed principle, i.e. the imaging device moves on top of the plants (see next section) to allow higher throughput and several measurements per day. In summary, a platform was required to allow non-destructive dynamic measurement of leaf area, and a precise recording of environmental conditions, in plants grown at densities reflecting the field conditions.

### Leaf conductance and response to VPD

In earlier studies, it was found that low canopy conductance (i.e. the amount of water transpired per unit of leaf area per unit of time) was an important adaptation to terminal drought stress in several semi-arid tropical crops, but this phenotype assessment depended on time-consuming measurements (Kholová *et al.*, 2012). One part of that phenotype assessment is the leaf area (described above). Another requirement is the rapid assessment of plant transpiration. Such measurements can be performed manually by gravimetrically determining transpiration in smaller experiments (e.g. Kholová *et al.*, 2012). A high throughput could be achieved by continuous assessment of plant transpiration of plants standing on scales, as it is done in the PHENOPSIS platform (Granier *et al.*, 2006). Finally, an important consideration in defining a suitable size for the platform was the type and number of genetic material that could be targeted for assessment (e.g. diversity panels for allelic variation, RILs/Fine Mapping/BCNAM populations for QTLs and breeding lines to speed up selection). Therefore, the platform was set outdoors, where the experimental conditions during the year (South India) cover a large VPD range and allows testing of many crops, from tropical to temperate species. Outdoors conditions were chosen mainly because of the difficulty of recreating changing VPD conditions in controlled conditions environments, whereas the light and VPD environment are more homogenous and easier to be followed outdoors. Notably, studying intra- or interspecific variations in crop water loss during the night is now possible, following recent results in wheat (Schoppach *et al.*, 2014). Other windows of research opportunities include the interaction between water use and the 3D architecture of the crop canopy, possible relationships between leaf movements during the day [especially in legumes or for example in *Arabidopsis* (Dornbusch *et al.*, 2012)] and patterns of plant water use, and of course the interplay between volumetric (leaf area expansion) and massic (transpiration) growth.

## The LeasyScan platform: 3D scanning and transpiration assessment

### How the technology functions

**Fig. 1.**
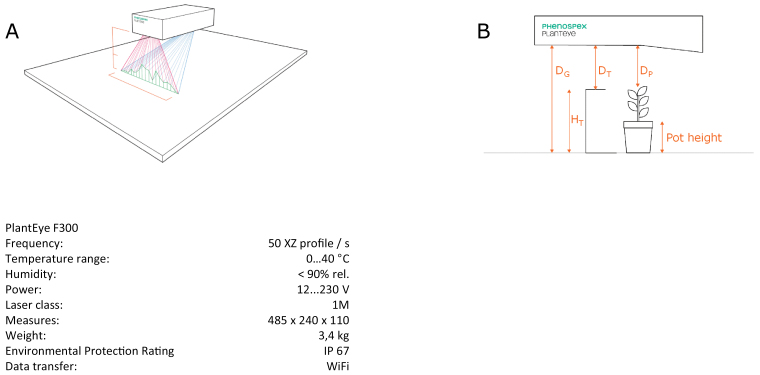
Schema of the scanning unit (PlantEye^R^). (A) How the 3D object (canopy; green) is reconstructed from the set of 2D images (50–80 images per second) of the reflection (red) of laser line (green) projected on canopy. (B) The distances (to the ground, D_G_, to the barcode target, D_T_, and to the plant, D_P_) that are used in the computation. T_H_ represents the target height and is used as a reference height for calculations. Pot height is set for the scanners and represents the height below which the data from the 3D data clouds are not used in the calculations.

The description of the requirements to assess traits altering plant water use in the previous section led to the development of a new platform, LeasyScan, whose principle was to have continuous and simultaneous monitoring of plant water use and leaf canopy development. In brief, the platform uses a set of scanners (PlantEye F300, Phenospex, Heerlen, The Netherlands), which are moved above the plants using a carrier device and generate 3D point clouds of the crop canopy, from which the leaf area and several other plant parameters are extracted after a segmentation process of the 3D data cloud (Fig. 1, scanner display).

The PlantEye sensor projects a very thin laser line in the near infrared (NIR) region of the light spectrum (940nm) on plants and captures the reflected light with an integrated CMOS-camera. NIR is used to increase the data quality, since all the light is reflected from plants. All artefacts from sunlight or background noise are automatically removed with intergraded optical- and algorithm-based sunlight filters. Moreover the sensor is temperature controlled, which allows the operation under full sunlight and environmental conditions of up to 45°C without any cutback or loss in data quality. During the scanning process the scanner linearly moves over the plants and generates 50 height profiles per second, those are then automatically merged into a 3D point cloud with a resolution of around 0.8×0.8×0.2mm into the xyz-direction, respectively. The measurements are triggered and stopped via mechanical barcodes (metal plates 20×50mm) positioned on the platform. Those barcodes are also used for calibration processes in order to define the distance from the scanner to the ground (Fig. 1). Thereby a high accuracy and reproducibility can be achieved independently of the carrier device. PlantEye computes a diverse set of plant parameters on the flight by meshing neighbouring points with a nearest neighbour search, similar to the method used by Fanourakis *et al.* (2014). From this triangle mesh a subsequent surface triangulation algorithm computes leaf area (which is the area of the leaf independently of its position and orientation in the 3D space and relative to the sensor), plant height and leaf angle distribution, which are all computed within a second.

**Fig. 2.**
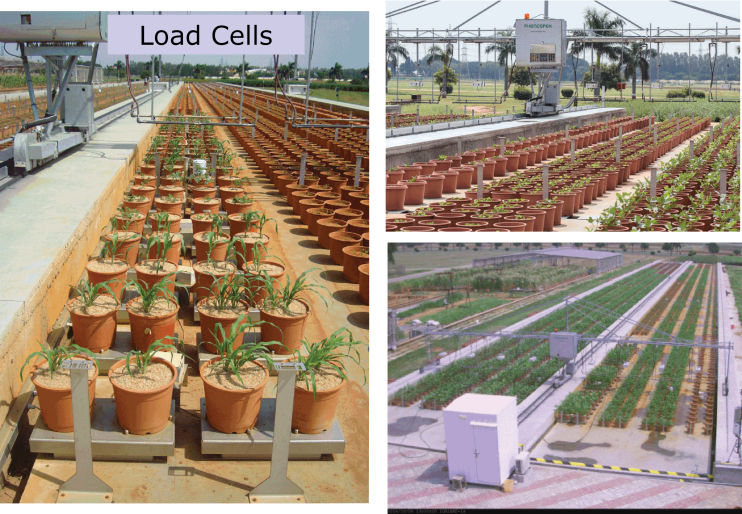
Phenotyping platform—LeasyScan. Scanner support device with eight scanners (PlantEye^R^) can assess the information from 3200/4800 sectors (60×60/60×40cm – a sector represents a replication unit and encompasses two pots) in 2h intervals with standard speed 50mm s^-1^; The platform length is 129.5 m and allows assessment of plant height, canopy size (3D area, projected leaf area) and canopy structure parameters (leaf angle).

The scanners are preset to image an area of 65cm width and a length of either 40 or 60cm, which is called a ‘sector’. The volume in which the 3D image is generated is then a cuboid of 65×40×100cm or 65×60×100cm. While extracting parameter data from the 3D image, the scanners are set to ignore point clouds that are below a certain height (pot height). Sector-wise binning of data point clouds is performed using a system of barcodes every 5 m to reset the scanner position in height and length. As in the lysimetric facility (Vadez *et al.*, 2014), our deliberate choice was to remain as close as possible to the field conditions and consider each of these sectors as a plot, in which plants are cultivated in each sector at a density similar to the field (for instance 24–32 plants per square metre for chickpea or 16 plants per square metre for pearl millet or sorghum). Therefore, each sector represents an experimental unit, and in the rest of the manuscript sectors may be called as such, or plots, or experimental units. The scanners are mounted on top of an irrigation boom, which is electronically controlled to be fully automated and speed-controlled. At a movement speed of 3 m min^-1^, eight scanners are capable of scanning 4800 sectors in slightly less than 2h. The speed and frequency of scanning can be adjusted depending on necessity—we currently operate at a rate of 12 scans per sector per day. The plant parameters that are measured by the scanners are the total leaf area (which is called 3D-leaf area), projected area (which is akin to the leaf area index, LAI), leaf angle (i.e. the average angle of vectors perpendicular to the surface of each triangulated unit, also called surface normal) and plant height (Fig. 2, the platform).

**Fig. 3.**
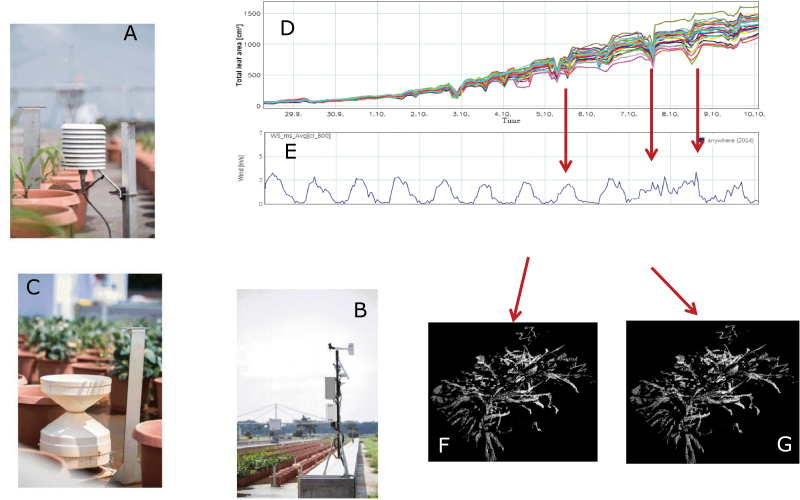
(A–C) Set of environmental sensors: (A) temperature, relative humidity, (B) solar radiation, wind speed, (C) rainfall. (D) Information on plant parameters in time visualized through web-based software interface (Hortcontrol^R^). Environmental data visualized in Hortcontrol, e.g. (E) wind. (F, G) 3D-point clouds accessed from Hortcontrol, at the LeasyScan platform. The Hortcontrol^R^ allows the basic data operations and quality control (e.g. data obtained during the windy part of the day (F) are of less quality compared to data obtained during windless part of the day (G) and are filtered for further analysis.

These parameters can be visualised through a web-based software interface (HortControl^R^), which allows the selection of sectors and performs basic grouping functions to assess how the experiment is progressing (Fig. 3, Sensors and HortControl display). In addition, the platform is equipped with a set of 12 environmental sensors (Campbell Scientific, Logan, Utah, USA) that continuously monitor relative humidity (RH%) and temperature (T°C), integrating values every 30min, one light sensor, one wind sensor and one rain gauge. Each scanner is wirelessly connected to a LAN through which the analysed data are downloaded onto a server, along with the 3D images. The environmental conditions can also be visualised in HortControl. 3D images are stored in the server and are accessible in HortControl. However, the system segregates the 3D data clouds from the analysed parameters and the weather information to keep the latter at a relatively small size. These 3D images can be reused at any time; for example, to recalculate new parameters based on a new algorithm for additional plant traits or for better optimised scanning software. Therefore, the scanning images become a repository of plant measurements, along with environmental metadata, that can be reused at a later date. Work is currently ongoing to perform a meshing of the 3D data cloud toward the segmentation of individual plant organs. An important factor to decide on in the scanning system was the signal noise ratio for our targeted phenotype (leaf area), and then check not only the resolution of the sensor itself but also the noise of the environment e.g. wind, diurnal rhythm of leaves, rain, reflection, conditioning the speed of the scanner movement and the number of images taken per second.

### Data visualisation in HortControl

All data gathered from PlantEye sensors, scales and associated climate sensors are stored in a central PostgreSQL database. The data can be accessed and visualized with the web-based HortControl software that allows follow-up of progress in the different variables that are measured (Fig. 3D–G). Data in this system are synchronized according to their time stamp with information of environmental conditions attached. In particular, at any time during the experiment the 3D image of any sector can be called for quality control (Fig. 3F, G), which is particularly useful to pinpoint possible outliers (for example in the case of sector-to-sector overlapping or other unexpected disturbance). It also allows the simultaneous plotting of the environmental conditions to the parameter evolution, for instance to qualitatively estimate reasonable wind thresholds for accurate canopy parameters assessment in each species. Scanning takes place every 2h, so that ~50 000 data points for each parameter are captured every day. This is in addition to the capture and integration of environmental data every 30min.

### Database access, processing and analysis

A major challenge of this platform, and of any high-throughput platform, is the extraction and analysis of the data. This issue was discussed in a recent review (Cobb *et al.*, 2013). At the same time, well-documented datasets represent a potential treasure trove to investigate plant growth processes on a large scale [for example in meta-analysis (Poorter *et al.*, 2010)]. In that regard, much focus was put on linking measurement data with the most critical environmental variables affecting plant growth (i.e. temperature, relative humidity and light).

To address these critical needs, tools have been developed to access data from the HortControl database. These data are queried from the database via an R-command library interface (R, version 4.2.1, the R foundation). Among the essential features of the library is a process for interpolating and filtering the data to reject outliers. For instance, wind affects the quality of the 3D images. Data obtained when the wind is too high to have useful information (from blurred images) should be filtered out (Fig. 3). Therefore, the filtering step allows discarding of data that were collected when the wind was above a user-defined threshold considered too high for good and steady 3D images (2 m s^-1^ is currently used as default threshold, although it depends on species and plant age). The filtering step also allows choice of date and time of day when data are extracted, and a time interval before and after the chosen time to calculate the median value in that interval. This step is quite important because wind varies during the day as well as leaf movements. Last, the scanning information is tagged to the timing of each scan, which is then linked to the environmental data provided by platform-attached sensors. The library also allows the extraction of the different weather variables that are collected on the platform, which are used for the calculation of VPD or thermal time.

Initially the platform will aim to assess the range of genetic variation in leaf area, transpiration and transpiration rate (i.e. canopy conductance) for mapping and screening purposes. The technological capacity of the platform would then compel a shift to analysing the data from a dynamic perspective; in particular, to decipher the response of leaf development patterns to environmental conditions, following earlier studies (see fig. 1 in Welcker *et al.*, 2011). In that respect, an alternative time stamp, right from the ‘R’ interface, is under development, which would be calculated from the temperature conditions and based on equivalent time at 20 degrees (Parent *et al.*, 2010). This feature would allow us to compare growth traces across experiments and analyse environmental effects on leaf area development, independent of temperature effects. In this way, the analysis will increasingly become an exercise of statistical treatment of time data series.

### Strength, weaknesses, and future opportunities and potential uses of 3D data clouds

The high throughput (~2400 scans per hour) of LeasyScan is a major improvement to the conveyor belt system, which is limited to about one imaging per plot/plant per day. This high throughput presents the prospect of following possible leaf movements in the course of the day, especially in the case of legume crops, which might be important in terms of water use. For instance, our preliminary observations in several legume species display clear hyponastic movements of leaves during the midday period, a tendency that is more pronounced in certain species like cowpea than in others like peanut. Whether these leaf movements have any relevance from a water use standpoint is unclear and we have here the technology to address these potentially important questions. Another of this platform’s strengths is its potential to capture simultaneously volumetric growth (the expansion of the leaf canopy) and massic growth (proxied by transpiration), and thus answer some critical questions on sink-source relationships in plants (Caldeira *et al.*, 2014, Tardieu *et al.*, 2014). To achieve this, proper filtering, smoothing and interpolation of the data is needed to generate interpolated hourly values of leaf area that match the hourly values of transpiration. A further strength is its ability to operate in outdoor conditions with plants that are grown at densities reflecting field conditions.

Of course, as any platform, there are a number of weaknesses. One is the plant age and leaf area up to which high quality scanning can be obtained and this was a key consideration for designing the platform. By and large, accurate scans in different species can be obtained up until a leaf area index (LAI) of 1.5 is reached (see section below). A detailed method analysis is in progress where these thresholds would be defined in several target crop types (Kholová *et al.*, unpublished). An improvement would consist of adding a scanner on the side to increase the resolution of the 3D data cloud. However the above-described phenotypes that determine overall plant water use are typically measured before reaching this LAI. In any case, leaf overlap is one limitation of LiDAR approaches, which also limits possible field applications to obtain relevant and precise information from thick canopies. Here, defining the phenotype well and then designing a way to obtain specific information from 3D images is an important part of overcoming possible limitations of the techniques, as Deery and colleagues did to measure the number of panicles from a wheat stand (Deery *et al.*, 2014). Because the wavelength used in LeasyScan is beyond the visible range, the 3D image provides no colour distinction that could proxy for water status or for senescence indices. However, the scanners have been designed in a way that other sensors could be slotted in if needed.

So far, the different variables provide an aggregate data point for all the plants contained in a given sector. Therefore, the current limitation, which is a work-in-progress and a tremendous opportunity, is the capacity to have a finer meshing of the 3D data cloud that would allow the segmentation of individual plant organs, especially leaves or branches. We think this is a critical avenue to focus on in the near future. In the case of cereals, this would allow assessment of tillering capacity, which is also known to be critical for setting plant water use (see van Oosterom *et al.*, 2011) but also leaf number and leaf size (Borrell *et al.*, 2014). The capacity to tiller is known to be under genetic (Kim *et al.*, 2010a) and environmental control (Kim *et al.*, 2010b), both of which could be characterized once the meshing of plants into individual organs is possible. In the case of the legumes, the branching pattern is also an important factor conditioning the port of the plant and then the leaf area and leaf area index, with expected important influence not only on water losses from the canopy but also from soil evaporation. For instance, preliminary observations from chickpea scans indicate that the projected leaf area, i.e. the area of soil that is covered by the leaf area (which is akin to the leaf area index), varies from the leaf area from a 3D image (3D-LA) in a genotype-dependent manner. In other words, genotypes with similar 3D-LA achieve different leaf area indices, which may have potential implication for soil evaporation. Also, different leaflet orientation in the 3D volume could have implications in terms of light interception.

## The LeasyScan platform: 3D scanning and transpiration assessment

### Relationships between destructive and 3D leaf area

**Fig. 4.**
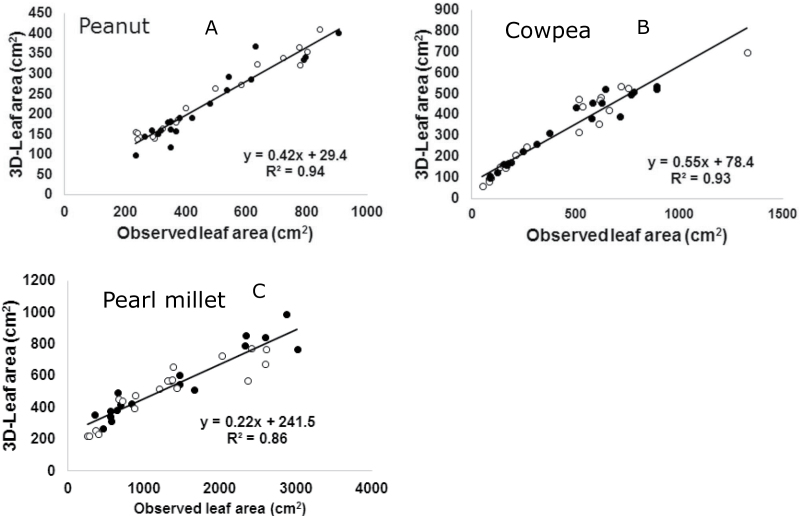
Validation experiments using the PlantEye^R^ technology prototype: leaf area of individual plants of (A) peanut, (B) cowpea and (C) pearl millet, in which leaf area was assessed destructively (observed leaf area) (Li3000, LICOR, Lincoln, Nebraska, USA) and compared to 3D-leaf area generated by the scanners, during different phases of plant development. For each plant species, two genotypes differing with the canopy structure were used (open and closed circles). In this experiment with a prototype scanner, the scanning width was only 32cm, compared to 65cm in the current plaftform (Fig. 5), which restricted resolution for pearl millet.

A prototype scanner was initially installed and tested to assess individual plants with a scanning width of 32cm, a length of 33cm and a maximum scanning height of 80cm. Although these parameters were restrictive, they were sufficient for testing, and were enhanced in upgraded versions of the scanner. The scanning width in the current setup is now 65cm, the length is either 40 or 60cm and the scanning height is 100cm. Validation was carried out with three species (peanut, cowpea and pearl millet) to reflect a wide range of canopy architecture, and two genotypes with putatively different leaf areas in each species. Plants were grown individually in 27cm diameter pots containing approximately 11kg of soil and held under optimal growth conditions. At regular intervals, until 6–7 weeks after sowing (depending on the crop), sets of plants were scanned, destructively harvested and leaf area was measured with a LI3000 leaf area meter (LICOR, Lincoln, Nebraska, USA). Overall, there was a very good fit between the observed leaf area data and the leaf area data derived from the scanner analysis of the 3D images (i.e. the 3D-LA) (Fig. 4, initial validation). The regression coefficient varied from 0.86 (pearl millet, Fig. 4A) to 0.93 (cowpea or peanut, Fig. 4B, C). It is notable that the scanners were able to reasonably estimate the leaf area of very large plants (leaf areas up to 3000cm^2^ in individual pearl millet plants). Even before reaching that size, the leaves were going beyond the sector perimeters, leading to some error between observed leaf area and 3D-LA, and explaining the lower R-square in the case of pearl millet. Since the data was gathered only by one perspective, the option exists for more accurate measurement by adding another scanner with a different perspective, the choice of this being a trade-off between the added precision on the phenotype versus the added cost (including computation and storage). The scanners are currently optimised and the fitness is similar to those presented here (Kholová *et al.*, unpublished).

**Fig. 5.**
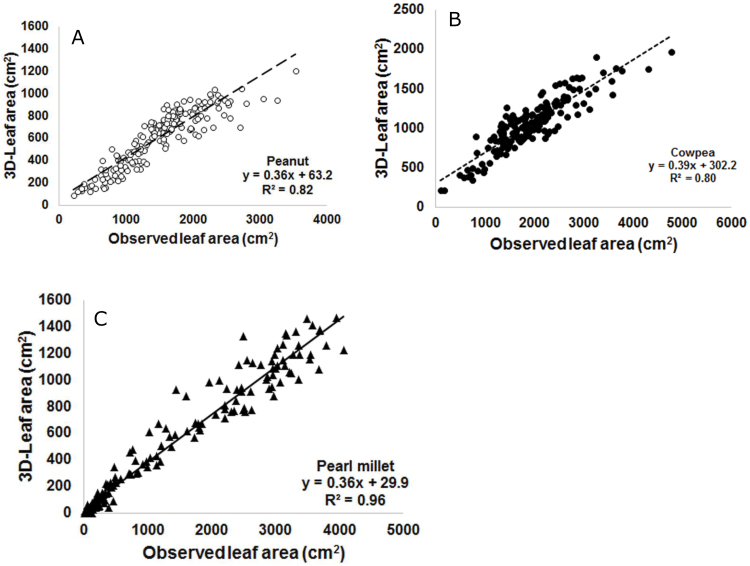
Repeated validation experiments using the LeasyScan platform: leaf area from sectors planted with peanut (white circles, dashed line), cowpea (black circles, dotted line) and pearl millet (black triangle, solid line) in field-like density (16 plants per square metre for pearl millet and 24 plants per square metre for peanut and cowpea) was assessed destructively (observed leaf area) (LICOR, Lincoln, Nebraska, USA) and compared to 3D-leaf area generated by the scanners, during different phases of plant development. For each plant species, three to four genotypes were assessed, all fitting the same regression line within each species (symbols not specified for genotypes).

A repeat validation experiment was carried out to compare destructive measurements of the leaf area to scanned data, using the platform setup described above and with plants growing at a density reflecting field conditions (24 plants per square metre for cowpea and peanut, 16 plants per square metre for pearl millet). Despite the expected level of overlap between neighbouring plants, the fitness was very high, i.e. 80–96%. This validation was a deliberate attempt to ‘push’ the system to its limit and assess the growth stage until which measurements could be reliably performed. The R-square values for peanut and cowpea were not as good as they were in the validation with individual plants (Fig. 4A, B), implying that we may have to decrease the plant density for these two crops to increase resolution. By contrast, the R-square for pearl millet was higher than in the individual plant assessment (Fig. 4C), likely in relation to the wider scanning (65cm), therefore reducing the effect of leaves going beyond scanning boundaries. Important considerations for the experiments were to clearly frame the conditions, timings, and data filtering standards per crop, allowing an accurate assessment of 3D-LA. The slope of the regressions between the 3D-LA and the observed leaf area differed somewhat between legumes and cereals. This indicated that the scanner revealed features likely associated with the 3D architecture of the crop canopy of these different crops families (Fig. 5). The slopes were different from 1, indicating that the 3D-LA predictions underestimated the observed leaf area, and this was more so as the plant size increased. However, within species, the different genotypes fitted the same regression line (Fig. 4; genotype detail not shown in Fig. 5) (more details in Kholová *et al.*, unpublished). Our interpretation is that despite a degree of overlap between leaves of the plants that the scanner cannot capture, this does not alter the comparison of the leaf area or the genotypic ranking, which was a critical necessity. The fact that the slopes differed between plant species suggests that the degree of overlap varies between species, very likely in relation to the 3D architecture of the canopy. Future research will be needed to assess whether the coefficient of overlap can be measured, whether it is repeatable over time for different crop species and whether such factors can be used to extrapolate 3D-LA to leaf area in each species. How this relationship alters plant-water relations is unknown, but suggests the possibility of linking the 3D architecture of the crop canopy to plant water use patterns or light interception. These were not initial targets for the development of this platform, but the technology offers the potential for new research questions that could become central to the future improvement of crop productivity.

### Canopy conductance

**Fig. 6.**
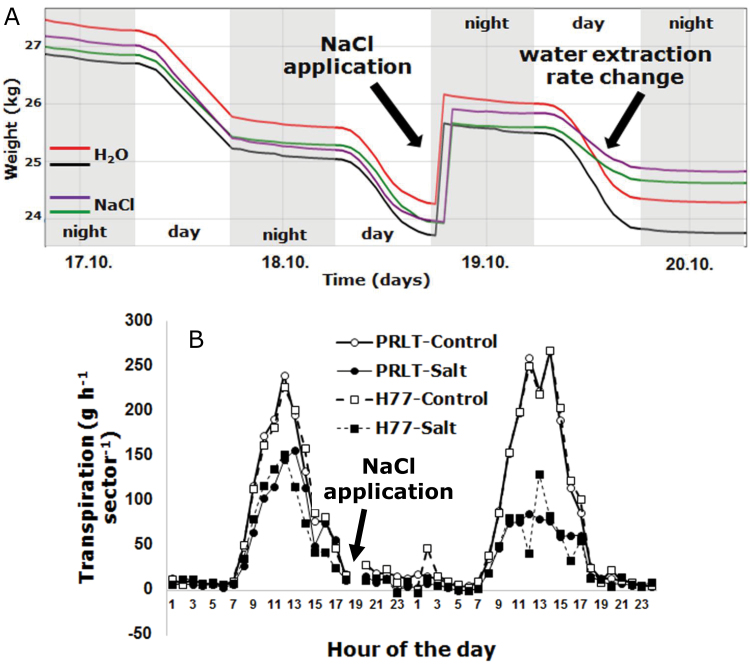
Data display of the analytical scales and transpiration data extracted from sector weights in an experiment where two genotypes of pearl millet were treated with NaCl on 18 October 2014 evening. (A) Typical trace of load cell weights, averaged across six sectors per treatment and genotype (untreated controls, red and black weight trajectory of genotype 1 and 2; salt treatment, green and purple weight trajectory of genotype 1 and 2). Before treatment the plants were kept under fully irrigated conditions. (B) Transpiration data before and after NaCl treatment in two genotypes of pearl millet (PRLT and H77). Data are the mean of six replicated sectors per genotype and treatment. (This figure is available in colour at *JXB* online.)

A basic idea in the development of the LeasyScan platform was to combine the measurements of leaf development parameters (which can be encapsulated in ‘volumetric growth’) with a continuous assessment of plant transpiration (or ‘massic growth’ considering transpiration as a proxy for photosynthesis), to obtain a continuous measurement of canopy conductance, based on earlier work (e.g. Kholová *et al.*, 2010; Zaman-Allah *et al.*, 2011), and a shift from earlier destructive measurements (Kholová *et al.*, 2012). Figure 6A demonstrates a typical trace of the evolution of the pot weight over time, before and after NaCl treatment, which further altered plant transpiration (visualized in HortControl). In this experiment, two pearl millet genotypes were cultivated in 27cm pots containing 12kg of Alfisol. Once plants were 10 days’ old, the pots were covered by a polythene sheet and a 2cm layer of low density polyethylene beads to prevent soil evaporation, so that pot weight differences would provide direct measurements of plant transpiration. There was a scanner setting of 65cm width and 60cm length. Each sector had two pots, and each pot two plants, giving a sowing density of 10 plants per square metre and each replication unit was 0.40 m^2^, with six replicated sectors for each genotype and treatment combination. The scales (Rugged Scale 50, Phenospex, Heerlen, Netherlands) have a capacity of 50kg, with 0.02% accuracy. The accuracy of these temperature-corrected scales (−10°C to +40°C range) was tested under artificial rapid increase in temperature (14°C h^-1^, i.e. much above our experimental conditions) and showed that the error remained within the stipulated 0.02% error range. The scales provided a reading with a 0.02% precision every second and these were integrated over one hour, giving readings with a precision of 0.1g. The treatment consisted of the application of 1 l of a 250mM NaCl solution, and the control received 1 l of non-salted water (Fig. 6). This was only a qualitative assessment and a proof-of-concept to assess how fast and accurate the system was able to detect changes in transpiration rates. In addition, these traces were important in the design of the type of interfacing scripts needed to extract meaningful transpiration information from numerous weight data. In any case, upon NaCl treatment the decrease in weight (the day following the treatment) was lower (Fig. 6A) and transpiration decreased more in the NaCl-treated plants of both genotypes than in the control plants (Fig. 6B). Similar results were obtained for two genotypes of sorghum, cultivated and treated in the same way (data not shown). Data filters are under development to segregate out weight changes due to watering or drainage. Therefore, the platform allows continuous and simultaneous measurements of plant development and transpiration within a time frame of an hour in an undisturbed manner and in the planting densities that reflect field conditions.

### Case studies: leaf area development dynamics

#### Fine-mapping recombinant inbred varying at three marker loci

Past research has identified a major terminal drought tolerance quantitative trait locus (QTL) on linkage group 2 (LG2) of pearl millet (Yadav *et al.*, 2002). The introgression of this QTL in the background of H77/833-2, a high tillering line and pollinator parent of a major hybrid for the driest pearl millet growing area of India, showed a yield benefit across several terminal drought stress environments (Serraj *et al.*, 2005). A dissection of the physiological traits underlying that QTL, and a mapping of these traits, pointed to differences in transpiration rate, in part as a consequence of differences in leaf area and tillering (Kholová *et al.*, 2012). Because the QTL interval was large, a high resolution cross was developed between the most promising of the introgression lines and the recurrent parent, and the F2 population (~2500 individuals) was genotyped using six polymorphic SSR markers (Sehgal *et al.*, 2012). Phenotyping of 160 individuals from this F2 population showing recombination has been carried out both in the field, in lysimeters and pot culture (Kholová *et al.*, unpublished) and here we tested a set of 33 most informative entries, selected from different phenotypic clusters (leaf area, biomass, transpiration efficiency, grain yield).

**Fig. 7.**
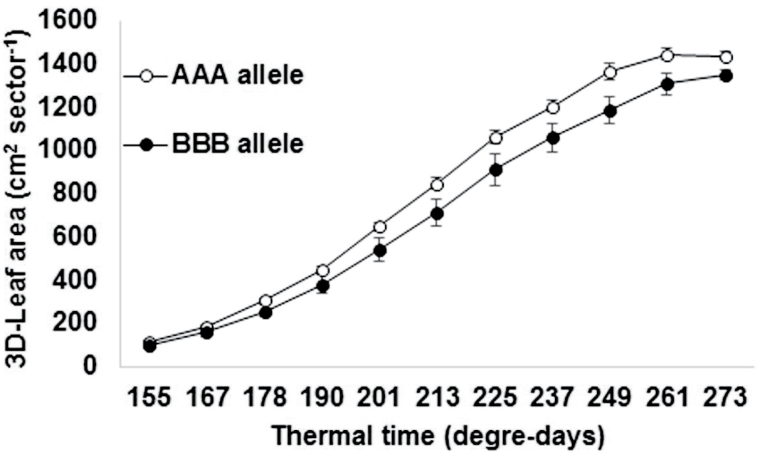
3D leaf area development dynamics within a 12 d period covering the 155–273 degree-days thermal time in pearl millet fine-mapping recombinants varying in parental allele at three marker loci within the terminal drought tolerance QTL region of linkage group 2 (Yadav *et al.*, 2002) (AAA, recurrent; BBB, QTL donor parent). Each data point for the AAA is the mean (±SE) of 10 lines. Each data point for the BBB is the mean (±SE) of 5 lines.

The materials were planted in the LeasyScan platform on 19 September 2014, using a sector dimension of 65cm width and 40cm length. Each sector included two pots of 27cm diameter filled with 11kg Alfisol collected from the ICRISAT farm. Three to four seeds were planted in four hills. Seedlings were thinned to one per hill 8 d after sowing and eventually to two seedlings per pot at 12 d after sowing. Therefore, each sector contained four pearl millet plants, giving a sowing density of approximately 16 plants m^-2^, typical of field populations. Four replicated sectors were used for each entry. The scanning started after the last thinning and the data are presented for the period 1–11 October. The calendar time was converted into thermal units taking a base temperature of 10ºC and optimal temperatures of 25–35ºC. Figure 7 compares the leaf canopy development pattern of 10 lines carrying the recurrent parent allele A at the first three loci within the QTL region (AAA) and of 5 lines carrying the QTL donor parent allele B at the first three loci within the QTL region (BBB). Here, recombinant containing the AAA allele had a more vigorous leaf area development than the BBB allele. Clearly, the leaf area development pattern of the two groups of lines differed and these differences could be pinpointed by the scan measurements. Measuring these differences destructively and manually would have implied major efforts. It should be noticed that the largest leaf area differences, i.e. at 249 degree-days after sowing, were no more than 13% indicating the capacity of the scanning technique to pinpoint small differences for fine genetic analysis. These leaf area differences may look small, but would have very large implications under water restricted conditions, as seen earlier (Kholová *et al.*, 2014). Possible immediate application is the mapping of the growth rate coefficients, and this can be applied to very large sets of entries. Of course, growth is a response to environmental conditions (e.g. Welcker *et al.*, 2011) and therefore repeated experiments with the same material over time under different evaporative demand would also allow us to compare the growth rate response coefficients to environmental conditions.

#### Pearl millet breeding lines adapted to different agro-ecological zones

Pearl millet is cultivated across a wide range of agroecological zones in India, delimited on the basis of annual rainfalls, i.e. the most arid A1 zone (receiving <400mm of annual rainfall and including the Western Rajasthan, parts of Haryana and Gujarat states), the A zone consisting of northern and north western India (receiving annual rainfall >400mm and includes the Eastern Rajasthan and parts of Haryana, Gujarat, and Uttar Pradesh), and the B zone consisting of peninsular Indian states (receiving annual rainfall >400mm and includes Maharashtra, Tamil Nadu, and Karnataka). A set of 97 breeding materials, including male sterile (B-lines), restorer lines (R-lines) and resultant hybrids (F1), developed for these different agroecological zones of India were used. These were respectively 14, 13 and 13 R-lines and F1 hybrids from the A1, A, and B zones respectively, and 4, 8, and 5 B-lines from the A1, A, and B zone respectively (several F1 had common male sterile B-lines). Because these materials have been bred for zones varying in the amount of rainfall, we tested whether the breeding resulted in both different patterns of leaf area development and different resulting leaf areas. The materials were planted in the LeasyScan platform on 19 September 2014, under the same conditions, procedures, and replications described above for the 33 high resolution recombinants.

**Fig. 8.**
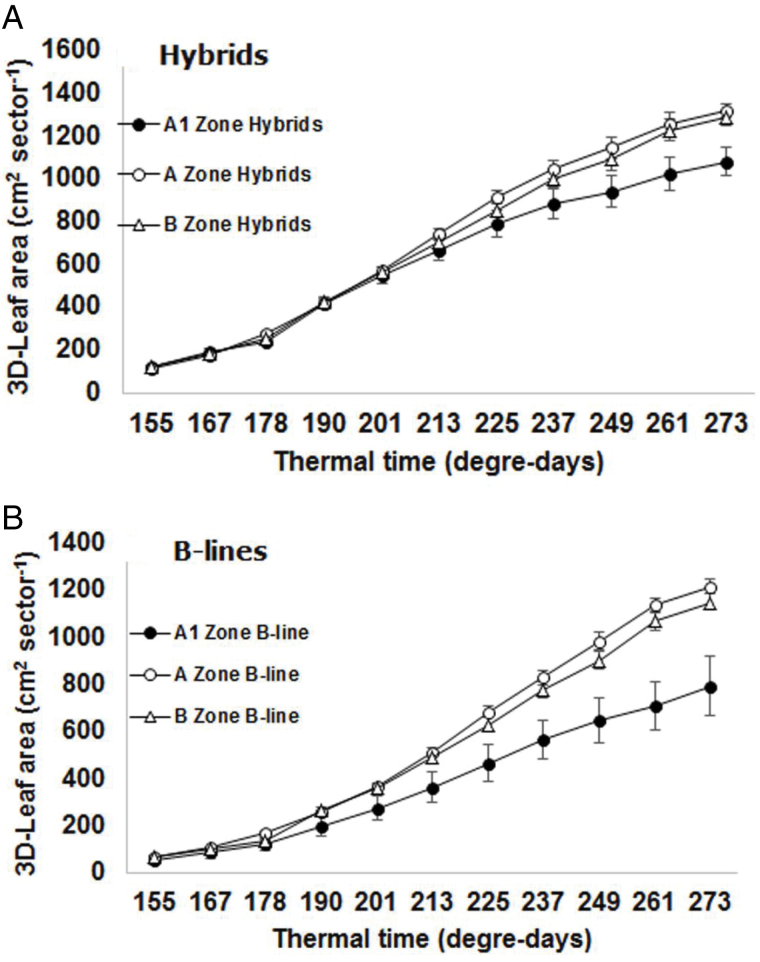
3D leaf area development dynamics within a 12 d period covering the 155–273 degree-days thermal time in (A) pearl millet hybrids and (B) B-lines, adapted to different agroecological zones of India ((A1, rainfall <300–400mm; A, rainfall >400mm in Northern states of India; B, rainfall >400mm for Peninsular states of India). Each data point for the hybrids is the mean (±SE) of 14, 13 and 13 hybrids for the A1, A, and B zone, respectively, and of 4, 8 and 5 B-lines for the A1, A and B zone, respectively.

Figure 8A shows that the leaf area development pattern of F1 hybrids bred for the A1 zone was dramatically different from those of F1 hybrids developed for either the A or the B zone. There were no significant difference between the leaf area development pattern of A and B zones. The range of variation, proxied by the size of the variation from the mean, in the A1 hybrids also shows that the variation among hybrids bred for the same zone was larger than for hybrids bred for the A and B zone. Even larger zone differences were found in the leaf area development pattern between B-lines bred for the A1 zone and those bred for the A and B zones. Similar but less striking variation was found for the R-lines (data not shown). Therefore, it appears clearly that materials bred for the A1 zones developed smaller leaf area as earlier discussed (van Oosterom *et al.*, 2003), in the order of 15% less for the F1 hybrids and in the order of 40% less for the B-lines. Early maturing hybrids (65–70 days to maturity) targeted for drought prone environments of the A1 zone indeed produce lower biomass in comparison to medium to late maturing hybrids (75–85 days to maturity) bred for relatively wetter A and B zones, hence the lesser leaf area in A1 hybrids was as expected.

### Assessment of the transpiration response to VPD conditions

Previous studies have shown the contrast between genotypes of pearl millet (Kholová *et al.*, 2010) and of sorghum (Kholová *et al.*, 2014) in the capacity to restrict transpiration under high VPD. These measurements have been so far performed mostly under controlled conditions and using individual plants cultivated in pots and with spacing wider than in field conditions. To our knowledge, there is only one study that has validated this trait in the field (Gilbert *et al.* 2011). The difficulty and limitation in the field was that transpiration rate data came from time consuming and highly variable porometric measurements. Here leaf area scanning and hourly assessment of sector water loss was combined to pinpoint putative transpiration rate response differences under conditions where plants are cultivated outdoors and in densities close to the field. For that purpose, the two contrasting pearl millet lines identified earlier were chosen (H77/833-2, VPD-insensitive; PRLT-2/89-33, VPD-sensitive) (Kholová *et al.*, 2010). Two contrasting sorghum parents used for the introgression of staygreen QTL were chosen (R16, VPD-insensitive; S35, VPD-sensitive) (Kholová *et al.*, 2012). The plants were grown under conditions similar to those described in the section above, except the planting was done on 10 September 2014. Thinning was done approximately at the same intervals as above, again resulting in two plants per pot (four plants per sector) at 12 d after sowing. The pots were covered with a polythene sheet and a 2cm layer of plastic beads applied on top of the sheet to prevent soil evaporation and scanning was started. The period presented here spanned between 24 September and 2 October, i.e. at 180–275 degree-days after sowing. This period was characterized by a mean maximum VPD of 2.46 kPa, ranging from 1.66 to 3.12 kPa.

**Fig. 9.**
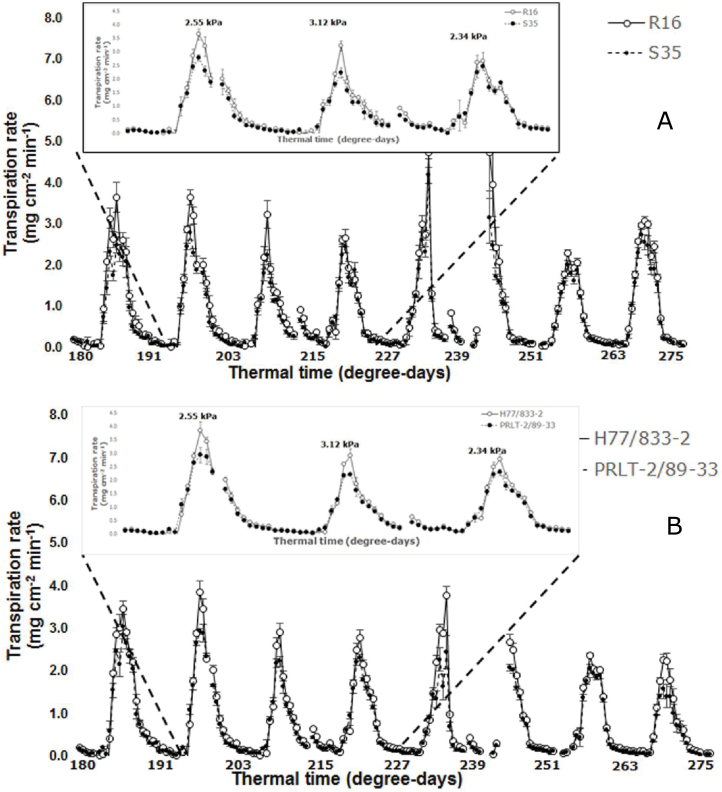
Transpiration rate profile (in mg cm^-2^ min^-1^) as a function of thermal time (degree-days, with base temperature of 10ºC and optimal temperature of 25–35ºC) in (A) two genotypes of sorghum (VPD-insensitive R16 and VPD-sensitive S35), and (B) in two genotypes of pearl millet (VPD-insensitive H77/833-2 and VPD-sensitive PRLT-2/89-33). The insert in each figure represents a close-up of a 3 d period at 191–227 degree-days. Each data point is the mean (±SE) of six replicated sectors for each genotype.

The transpiration pattern of R16 and S35 over this period showed the usual transpiration rate peak around the midday period. Quite consistently across days, the transpiration rate of VPD-insensitive R16 was higher than in VPD-sensitive S35 (Fig. 9A). The Fig. 9A insert shows in more details the transpiration rate over three consecutive days (i.e. at 191–237 degree-days), and indeed shows a consistent pattern of having transpiration rate in R16 above that in S35 for about 2–3h during the midday period. Similar results are shown for the two pearl millet genotypes (Fig. 9B). Here also, the transpiration rate of VPD-insensitive H77/833-2 was consistently above that of VPD-sensitive PRLT. The Fig. 9B insert shows the details of the transpiration pattern over three consecutive days. These results then provide experimental support to the theoretical representation of that mechanism (see fig. 1 in Sinclair *et al.*, 2005). Similar kind of data was also obtained in pairs of lines of cowpea and peanut (data not shown). Several QTLs for the capacity to restrict transpiration under high VPD have been mapped in pearl millet (Kholová *et al.*, 2012), three of which were co-mapped with the yield-based terminal drought tolerance QTL found earlier (Yadav *et al.*, 2002). This work was done by manually phenotyping plant transpiration and leaf area, with the limitation that the destructive measurement could only provide one snapshot of the plant response. LeasyScan provides the opportunity to access many more comprehensive details on the effects of VPD on both the response of the volumetric growth (leaf growth rate) and massic growth (transpiration, taken as a proxy for photosynthesis). We have shown the physiological significance of the transpiration response to VPD across crops (Vadez *et al.*, 2014). The current LeasyScan setup therefore allows VPD response measurements at a very large scale in a seamless manner and in conditions that are close to field conditions. Optimization of the analytical scale data treatment is still needed to filter out erroneous transpiration data (for instance following rain or irrigation). Expansion in the analytical scale capacity is currently ongoing.

## Conclusion

In order to decipher the possible causes of important phenotypes associated to better adaptation to water limitation of several semi-arid tropical crops, a phenotyping concept associating 3D scanning of plant leaf area and gravimetric measurement of plant transpiration with analytical scales, in plants cultivated at densities reflecting the field conditions, was developed. This concept provides a high-throughput capacity to measure small differences of both leaf area development and fine transpiration rate in the course of the day. The possibility of scanning each experimental unit (sector) at least 12 times a day creates the opportunity of measuring leaf movements and their possible importance for plant water use. Great opportunity lies in meshing of the 3D data cloud toward the identification, and possible follow-up over time, of individual plant organs, in particular branches and tillers.
